# Effects of Lead on Ultrastructure of *Isoetes sinensis* Palmer (Isoetaceae), a Critically Endangered Species in China

**DOI:** 10.1371/journal.pone.0139231

**Published:** 2015-09-25

**Authors:** Guohua Ding, Chunye Li, Xu Han, Chunyu Chi, Dawei Zhang, Baodong Liu

**Affiliations:** 1 Key Laboratory of Plant Biology in Colleges of Heilongjiang Province, Harbin, 150025, P. R. China; 2 College of Life Science & Technology of Harbin Normal University, Harbin, 150025, P. R. China; 3 Modern Experimental Center of Harbin Normal University, Harbin, 150025, P. R. China; Youngstown State University, UNITED STATES

## Abstract

*Isoetes sinensis* Palmer (Isoetaceae) is a critically endangered fern that is a marsh plant (that is an aquatic or amphibious plant) in China. To evaluate damage or influence of lead (Pb) on cell ultrastructure in *I*. *sinensis*, we used 2000mg·L^-1^ Pb(NO_3_)_2_ solution to treat *I*. *sinensis* for 35d, and used transmission electron microscope (TEM) to observe the cell ultrastructure of leaf blades and roots of the plant. Our results indicated that Pb induced distinct changes of the organelles including chloroplast, mitochondria, nucleolus and vacuole. The level of damage organ was lower leaf > upper leaf > root The typical performance of the damages caused by lead shown that part of the nucleolus cracked; the cristae dilated, matrix vacuolized and membrane structure blurred in mitochondria; the vacuole cracked; grana lamella decreased, stroma lamella loosed, starch grains decreased, and membrane structure was disrupted in chloroplasts; Pb deposits were present on cell wall. The damages to chloroplasts and mitochondria were relatively severe, while damage to the nucleus was relatively lighter. The damage to the cell ultrastructure of leaf blades with direct contact with Pb was more severe than that without direct contact with Pb.

## Introduction

Lead (Pb) is one of the major heavy metal pollutants in the world. It can be transported inside plants through absorption by the root and leaf blade, and accumulate in the root, leaf blade and seed [1]. Through food chain, Pb accumulates in the human body and does damage to the nervous, circulatory and digestive systems. In severe cases, it can cause sudden death [2]. In the past 50 years, global Pb emission in the environment reached 783,000 tons [3]. As the largest Pb producing and consuming country, Pb-relevant enterprises in China usually lagged behind production technology and used low-tech equipment, causing Pb waste, harm to the environment, and creating a danger to human health [4].

*Isoetes sinensis* Palmer (Isoetaceae) is a critically endangered first-class protected plant in China [5]. With the rapid development of industry and agriculture, a large amount of industrial wastewater, waste gas and pesticides are emitted into the environment, leading to damage of the remaining *Isoetes* habitat. With the danger of genetic homogeneity and competition with accompanying species, *I*. *sinensis* is under imminent threat of extinction [5–8]. However, there has been no research conducted on the impact of heavy metal pollution on *I*. *sinensis*, especially on ultrastructural effects. This paper used a transmission electron microscope (TEM) to study the influence of Pb on the cell ultrastructure of *I*. *sinensis* and aims to provide further evidence of the damaging mechanism of Pb to the cell structure of *I*. *sinensis*.

## Materials and Methods

### Plant material and cultivation

Material from *I*. *sinensis* originated from the Huading Mountain in Zhejiang Province in 2001. Material was cultivated in a greenhouse at Harbin Normal University.

Soil for cultivating the plants was derived from hilly land topsoil (5cm–20cm) in Yabuli town of Heilongjiang Province. After air-drying, the soil was passed through 2mm sieve and mixed with water into clay for planting. *Isoetes sinensis* grew in a greenhouse at 18–25°C during March and April of 2013. When the plants were 20cm tall, selected 21 plants and transplanted each three plants into a cuboid plastic container of 11cm by 9cm by 13cm with 650g of soil from Yabuli. Each container was put into a 3,000ml beaker, in which 2,200ml of distilled water was added for submerging half of an adult blade of *I*. *sinensis*.

Although the plants we studied indeed belong to endangered and protected plants, they were cultured by our-self since the material had been given by others. So no specific permissions were required for the plants. In addition, I confirm that our study does not require a permission to use soil for cultivating plants, because the soil belongs to the individual of Baodong Liu.

### Pb stress treatment

After an acclimation period of one month, the cultivated *I*. *sinensis* recovered from transplanting and were randomly divided into two groups. The first group with 12 plants was considered treatment group, in which 2200mL of 2000mg·L^-1^ Pb(NO_3_)_2_ solution was added to each beaker to replace distilled water. The second group with 9 plants continued with the distilled water and served as control group. During the treatment period, *I*. *sinensis* was kept in light for 12h, the photophase temperature was set 20–25°C, the scotophase temperature was set 18–20°C and the relative humidity was 60%–70%. Every day, deionized water was added into the beaks to keep the total volume of Pb(NO_3_)_2_ solution unchanged in order to keep the concentration of Pb unchanged. After 35d, *I*. *sinensis* was harvested and separated into root, rhizome, upper leaf (no contact with water or Pb solution, over water or solution) and lower leaf (contact with water or Pb solution, in water or solution) for the observation of the cell ultrastructure.

### Ultrastructure observation

Fresh samples (segments of root and leaf) of *I*. *sinensis* were harvested and quickly put into 2.5% glutaraldehyde, and refrigerated at 4°C for two days for pre-fixation. Leaf sample was extracted air by using injector in advance for immersing. A phosphate buffer of pH7.2 was used to wash the fixed material three times for 10 minutes each time. The samples were fixed again in 2% (v/v) osmium tetroxide for 1.5h and washed with phosphate buffer of pH7.2 three times again for 10min each time, and then step-by-step dehydrated in graded ethanol solution of 50%, 70%, 80%, 90% and 100% concentrations and each time lasted for 10–15 min. The dehydration in the ethanol of 100% concentration was made three times. Substitution was made once using a mixture of acetone and absolute ethyl alcohol 1:1 (v/v), and once more only using acetone. Each substitution lasted 10min. The samples were embedded in a mixture of acetone and Epon812 with 1:1, 1:2 and 1:3 for 1h, 2h and 2–3d, respectively. Finally, the samples were put into a constant temperature incubator for polymerization after 17h at 40°C, 24h at 45°C and 24h at 60°C. An ULTRACUT-E ultramicrotome (Reichert Ultracut R, Leica, Vienna, Austria) was used for slicing and a Hitachi H-7650 TEM (Japan) was used for observation and photographs.

## Results

### Pb causes ultrastructural changes in root cells of *I*. *sinensis*

**Fig 1:**
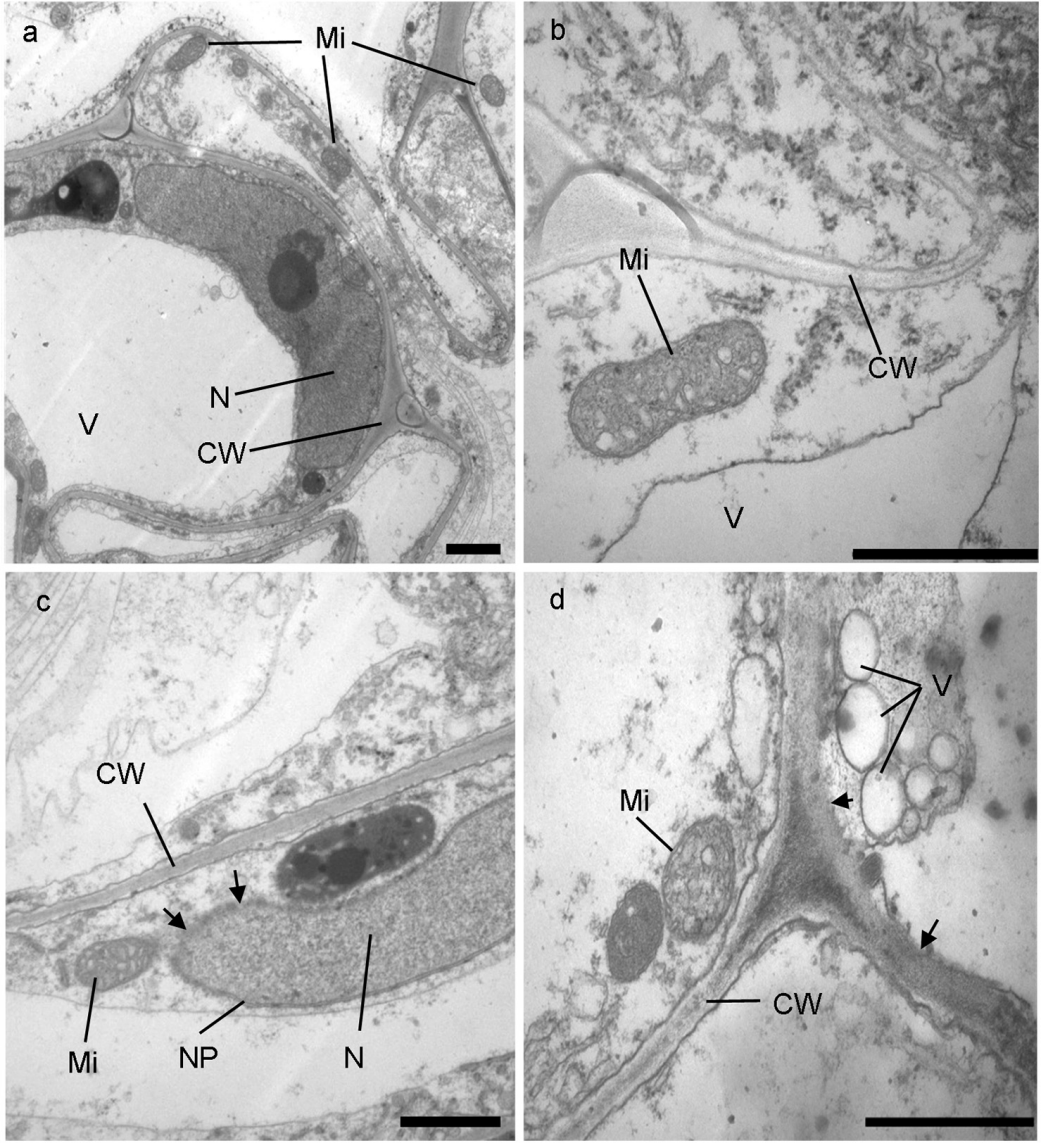
Transmission electron micrographs (TEM) of cell ultrastructure in roots of *I*. *sinensis*. Large vacuole, elonged mitochondrion and distinct nucleus in control samples (a, b). Little changes of shape of mitochondrion (c,d), nucleoplasm (c), cell wall (d) and small vacuole (d) in Pb treated samples. Bars = 1 μm. V-vacuole; Mi-mitochondrion; CW-cell wall; N-nucleus; PM-plasma membrane; NP-nucleoplasm.

In the control group, the double membrane structure of cell nucleus in root was clear, chromatin was evenly distributed, the nucleolus was complete and the boundary to nucleoplasm was distinct and clear; mitochondria were circle or oval shaped and the double membrane was clear, with obvious cristaes; the vacuole was large and complete (Fig 1A and 1B) and the structure of the cell wall was complete and the surface was smooth, without adhesion of particle Pb. In the treatment group, after five weeks in 2000mg·L^-1^Pb(NO_3_)_2_ solution, the morphological structure of the nucleus in the root cell of *I*. *sinensis* retained its integrity and chromatin was evenly distributed but the nucleoplasm became partly dimmed (as indicated with the triangular arrowheads in Fig 1C). The mitochondria were not injured obviously but double membranes of some mitochondria dimmed and cristaes lightly swelled, even some mitochondria changed their shape from elongated to oval or rounded and the inner disarranged in a messy (Fig 1D). There were no significant differences in the cell walls but the vacuole fractured into many small vacuoles and plasma membranes were partly damaged (Fig 1D, as indicated with the triangular arrowheads). Thus, it can be seen that Pb damaged some root cells of *I*. *sinensis*. However, the organelle structures of most cells were normal. On the whole, the damage on root caused by Pb stress was light.

### Pb caused ultrastructural damage in upper leaf blade cells of *I*. *sinensis*

**Fig 2:**
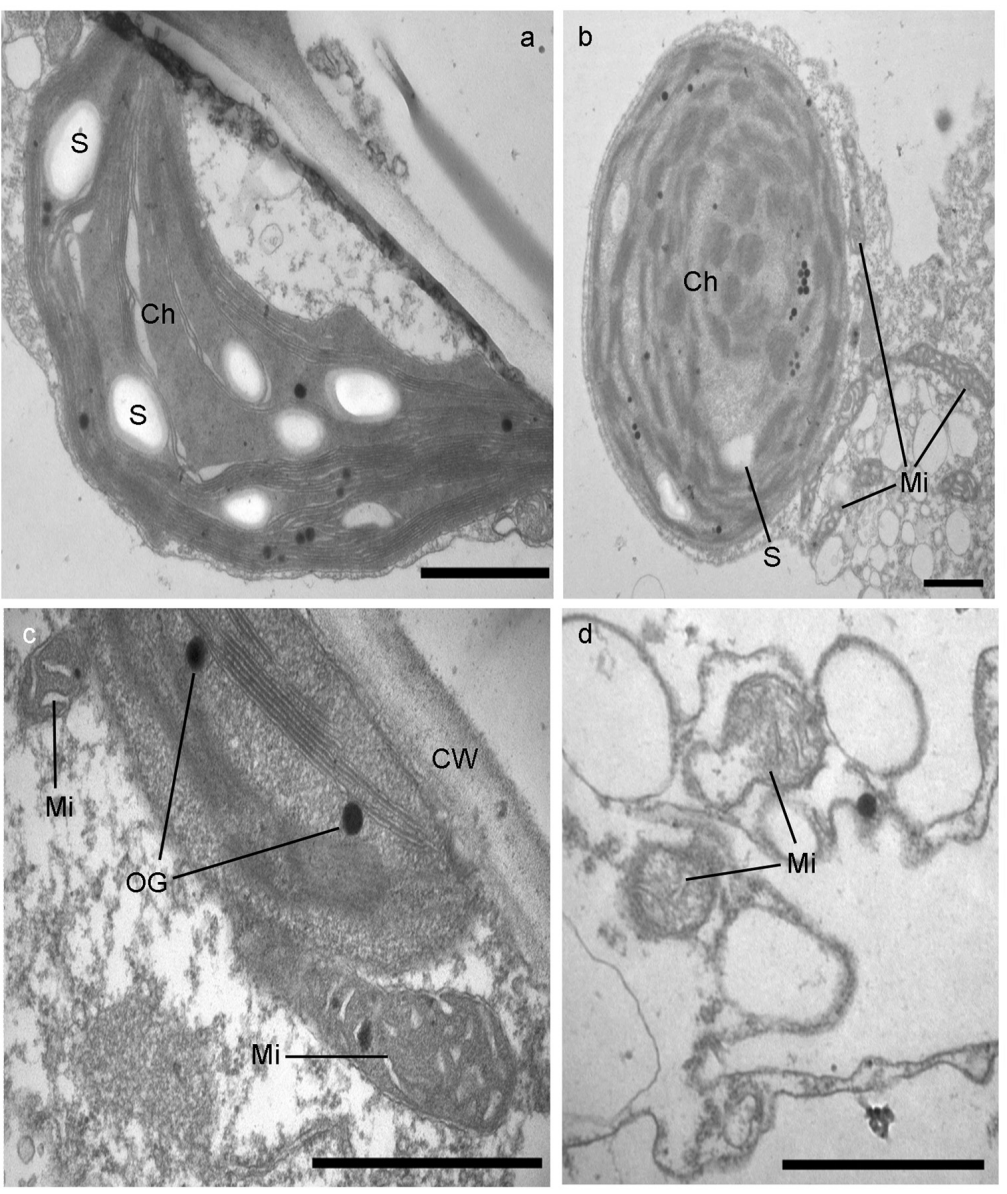
Transmission electron micrographs (TEM) of cell ultrastructure in upper leaves of *I*. *sinensis*. Spindle-shape chloroplast with many starch grains and integrated lamella in control samples (a). Swollen (b) and normal (c) chloroplast, various shapes of mitochondria (c, d) in Pb treated samples. Bars = 1 μm. Mi-mitochondrion; Ch-chloroplast; CW-cell wall; S-starch grain; OG-osmiophilic globule (plastoglobuli); PM-plasma membrane.

There was no great difference in the cell nucleus of an upper leaf blade in *I*. *sinensis* between the control group and the treatment group. Both had clear double membrane of mitochondria, compact and complete nucleoli and evenly distributed chromatin. In the cells of the control group, chloroplasts performed spindle-shape with clear double membrane structure, contained undisturbed lamellar system with more starch grains and few plastoglobuli (Fig 2A). The double membrane structure of mitochondria was clear, the cristae were obvious and the surface of the cell wall was smooth and there were no particles of Pb on it. After five weeks of treatment with 2000mg·L^-1^Pb(NO_3_)_2_ solution on the cell of upper leaf blade of *I*. *sinensis*, chloroplasts in some cells (about 30%) swelled and showed oval or round shape, in which number of starch grains markedly reduced but a few plastoglobuli appeared, while the structure of thylakoid maintained normal with distinct and complete lamellar system (Fig 2B and 2C). No changes in the cell wall structure between two groups strikingly exhibited (Fig 2C). However, the morphology of some mitochondria changed, such as one end dilated, the inner vacuolized, and the cristae disappeared (Fig 2D). The influences of Pb stress on the cell nucleus and cell wall in the upper leaf blade of *I*. *sinensis* was small and negligible, while the influence of Pb on chloroplasts and mitochondria were larger.

### Pb caused ultrastructural damage in the lower leaf blade cell of *I*. *sinensis*

**Fig 3:**
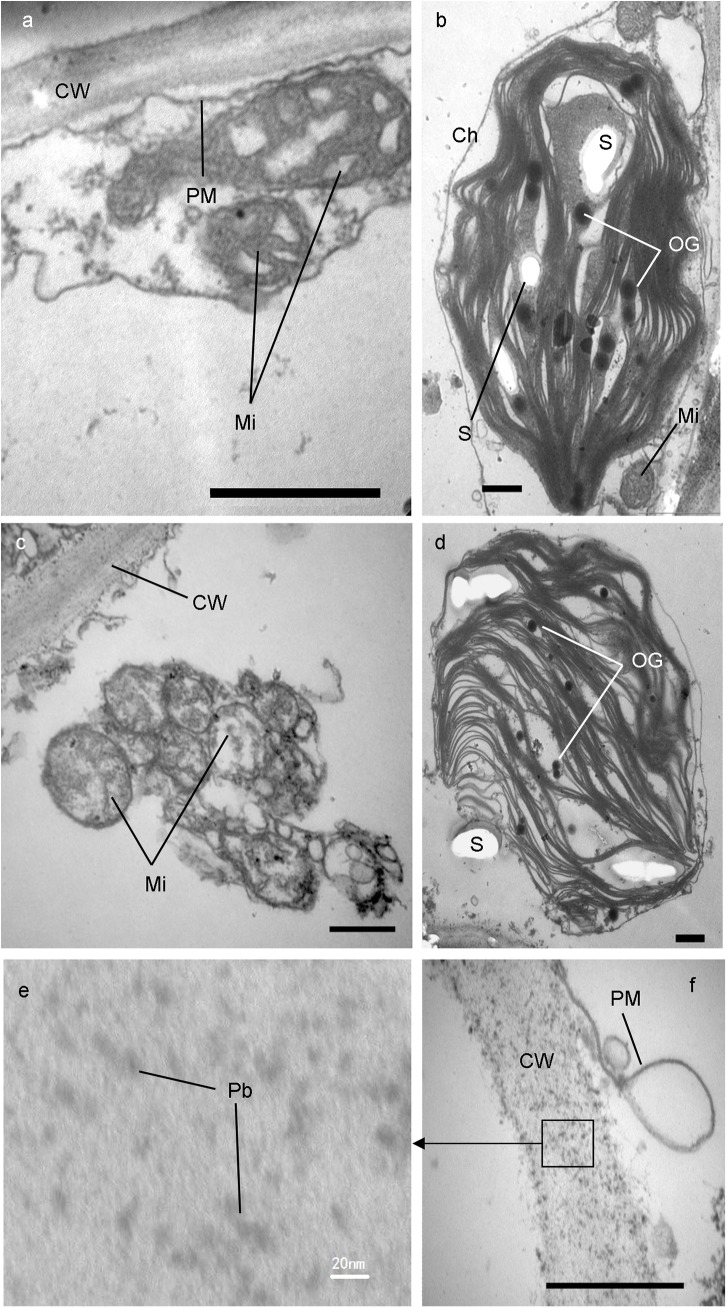
Transmission electron micrographs (TEM) of cell ultrastructure in lower leaves of *I*. *sinensis*. Smooth cell wall and unchanged mitochondria in control samples (a). A slight damaged chloroplast with loosely arranged lamella and many plastoglobuli (b), serious damaged mitochondria (c) and disrupted plasma membrane (f) and deposited Pb particles on cell wall (e, f) in Pb treated samples. Bars = 500 nm. Mi-mitochondrion; Ch-chloroplast; CW-cell wall; S-starch grain; OG-osmiophilic globule (plastoglobuli); PM-plasma membrane.

Using TEM, it was observed that in the cells of lower leaf blade of *I*. *sinensis* in the control group, chloroplasts were more abundant and were located near the edge of the cells; they showed spindle-shape with clear double membranes, typical organization of grana and stroma thylakoid, and many prominent starch grains. Mitochondria were more plentiful and were evenly distributed at the edges of the cells and around the chloroplasts, they were sphere or oval shaped and their double membrane structure was clear. The tubular cristaes were evenly distributed in the mitochondria. The surface of cell wall was smooth, the thickness was even and the plasma membrane was close to cell wall (Fig 3A). In the cells of treatment group, most chloroplast swelled to sphere-shape instead of spindle-shape, grana lamella were reduced, stroma lamella loosely arranged, the starch grains were fewer but the plastoglobuli were more, the double membrane was slightly disrupted, and a small amount of contents had begun to overflow from chloroplasts (Fig 3B). Some chloroplasts presented their closed envelope completely cracked and released their contents to the surrounding cytoplasm, finally disintegrated themselves (Fig 3B and 3D). Mitochondria decreased significantly and gathered. Both inner and outer membrane of them dilated and mixed together so closely that difficult to distinguish the double structure. The cristae of the mitochondria disappeared. Their matrix became turbid, in which some vacuoles presented (Fig 3C). On the cell wall, there were many small Pb particles deposited, which size went from a few nanometers to 10 nanometers. The plasma membrane was severely disrupted (Fig 3E and 3F).

## Discussion

The results of this study show that the degree of damage to the lower leaf blade of *I*. *sinensis* was larger than that of the upper leaf blade and the root, and are directly due to the treatment method of this experiment, in which the lower leaf blade and the roots directly contacted with Pb. According to our test data, the Pb concentration in the environment of the lower leaf blade was larger than that of root. Thus, the cell damage to the lower leaf blade was the most severe. In a cell of samples contacted with Pb, the damages to the mitochondria and chloroplast were larger than to the cell nucleus. The damage to the cell nucleus mainly manifested in the break-up of the nucleolus and the agglomeration of chromatin, which may be due to that the combination of heavy metal ions and nucleic acid and other macromolecule substances in nucleus resulted in an agglomeration of DNA molecule and further damaged the cell nucleus [9].

Reduction of starch grains, increase of plastoglobuli, disruption of envelope, changes of chloroplasts shape and alteration lamella structure were rather obvious in lower leaf blade of *I*. *sinensis* exposure to Pb, which are similar to results of some researchers[10–12]. The reason of starch grains decrease is possible that the chloroplasts were Pb-sensitive and severely suffer from Pb, which influenced photosynthesis and synthesis of organic matters.

The mitochondria expanded, inside and outside membranes disintegrated, their cristaes disappeared and the inside vacuolized. It is noteworthy that a large number of the mitochondria gathered and vacuolized in the lower leaf blade of *I*. *sinensis*.

The cell wall is the first barrier to prevent heavy metals from entering into plant cells and has strong capacity of adsorption and accumulation to positive ion. Liu and colleagues studied pea (*Pisum sativum* L.) seedlings exposure to Pb and found that there were some Pb particles depositing on the inside of cell walls and the plasma membranes closed to cell wall were damaged[13]. We also found that after exposure to Pb, the plasma membranes in the lower leaf blade of *I*. *sinensis*, which was close to cell wall, was drastically disrupted. Not only on the inside of the cell wall, but also on the whole cell wall, there were many black Pb particles from a few nanometers to ten nanometers deposited, this may be due to loose of cell wall and expansion of whole cell induced by heavy metal stress [14]. The loosened cell wall structure tends to absorb more Pb particles. The fact that Pb particles deposited on cell walls was also found by some researcher [12, 15, 16].

Through TEM, we observed that the damage level of every organelle in every cell of the same sample was different. The damages to mitochondria and chloroplasts were severer while that of nuclei were lighter. Even in the same cell the damages to the same organelles, for example mitochondria, also were various. In the same cell of *I*. *sinensis* exposed to Pb, some mitochondria appeared complete double envelope structure and clear cristaes, whereas some mitochondria showed sphere shape with disrupted structure in a mess, or with vacuoles inside. The difference in the damages among organelles in the same cell could be associated with self-protection of plants. Because aged mitochondria adsorb easily heavy metals to damage themselves, which reduces the damage of Pb to other mitochondria and further maintains the normal physiological function of *I*. *sinensis* exposed to lead. This may play a key role in improving Pb tolerance in *I*. *sinensis*.
